# Effect of yoga on quality of life of CLBP patients: A randomized control study

**DOI:** 10.4103/0973-6131.66773

**Published:** 2010

**Authors:** Padmini Tekur, Singphow Chametcha, Ramarao Nagendra Hongasandra, Nagarathna Raghuram

**Affiliations:** Division of Yoga and Life Sciences, Swami Vivekananda Yoga Research Foundation (SVYASA), Bangalore, India

**Keywords:** Chronic low back pain, flexibility, quality of life, stress, yoga

## Abstract

**Context:**

In two of the earlier Randomized Control Trials on yoga for chronic lower back pain (CLBP), 12 to 16 weeks of intervention were found effective in reducing pain and disability.

**Aim:**

To study the efficacy of a residential short term intensive yoga program on quality of life in CLBP.

**Materials and Methods:**

About 80 patients with CLBP (females 37) registered for a week long treatment at SVYASA Holistic Health Centre in Bengaluru, India. They were randomized into two groups (40 each). The yoga group practiced a specific module for CLBP comprising of asanas (physical postures), pranayama (breathing practices), meditation and lectures on yoga philosophy. The control group practiced physical therapy exercises for back pain.

Perceived stress scale (PSS) was used to measure baseline stress levels. Outcome measures were WHOQOL Bref for quality of life and straight leg raising test (SLR) using a Goniometer.

**Results:**

There were significant negative correlations (Pearson’s, *P*<0.005, r>0.30) between baseline PSS with all four domains and the total score of WHOQOLBref. All the four domains’ WHOQOLBref improved in the yoga group (repeated measures ANOVA *P*=0.001) with significant group*time interaction (*P*<0.05) and differences between groups (*P*<0.01). SLR increased in both groups (*P*=0.001) with higher increase in yoga (31.1 % right, 28.4 % left) than control (18.7% right, 21.5 % left) group with significant group*time interaction (SLR right leg *P*=0.044).

**Conclusion:**

In CLBP, a negative correlation exists between stress and quality of life. Yoga increases quality of life and spinal flexibility better than physical therapy exercises.

## INTRODUCTION

In recent years, quality of life (QOL) has become a key concept in the medical community where health care places dual emphasis on treatment and quality of care. The World Health Organization (WHO) defines QOL as an ‘individual’s perception of his/her position in life in the context of culture and value system in which they live and in relation to their goals, expectations, standards and concerns’.[1] It depends on a patient’s physical, psychological and social responses to a disease and its treatment.[2]

One per cent of the US population is chronically disabled due to CLBP.[3] Studies on QOL in chronic diseases including CLBP point to factors such as chronicity, seriousness of the episode, stress and depression that reduce the QOL.[4] CLBP in women seems to be associated with the lowest quality of life amongst many types of non-malignant chronic pains as was observed in a survey carried out in a multidisciplinary pain clinic in Netherlands.[5]

In CLBP, the reduction in quality of life could be attributed to sleep disturbances, fatigue, medication abuse[6] functional disability[7] and stress. Amongst these, psychological factors such as depression, anxiety, fear and anger seem to have a greater impact than biomedical or biomechanical factors on CLBP related disability and QOL.[8] Regression analysis in a group of 1208 chronic pain patients showed that pain catastrophizing had stronger association with quality of life than the intensity of pain.[5] Similarly, in patients with fibromyalgia[9] and CLBP,[10] the degree of pain, perceived disability and QOL were influenced more by their mental health status than the degree of physical impairment.

Multidisciplinary biopsychosocial rehabilitation has been shown to be better than usual care in improving QOL with reduction in pain and functional disability in patients with chronic back pain.[11] Yoga, with its holistic approach to improve overall quality of life, offers several self regulatory practices that aim at correcting these psychological factors that contribute to low QOL. An integrated approach to yoga therapy (IAYT) that includes practices at physical, breathing, mental, intellectual and emotional levels has been shown to be effective in improving the QOL in several chronic conditions such as fibromyalgia,[12] rheumatoid arthritis[13] and cancer.[14] Our recent randomized control study on patients with CLBP has shown that an intensive residential short term program of IAYT can reduce the intensity of pain and disability and improve spinal flexibility.[15]

### Objective

This study was planned to: (i) compare the effect of yoga with physical therapy exercises on QOL in patients undergoing a short term intensive residential program for CLBP (ii) study the baseline correlations between QOL and stress scores.

### Hypothesis

QOL in yoga group would be better than control (physical exercise) as yoga is a multi-dimensional treatment modality that caters to all the levels of existence.

## MATERIALS AND METHODS

### Subjects

A total of 160 patients with CLBP admitted to a holistic health home in Bengaluru (South India) from April 2005 to June 2006 were screened. Of these, 80 who satisfied the inclusion criteria were recruited. Statistical calculation using G power software yielded a sample size of 35 per group for an effect size of 0.89 (calculated from our earlier interventional pilot study) with an alpha at 0.05 powered at 0.8. The inclusion criteria were (a) history of CLBP of more than three months (b) pain in lumbar spine with or without radiation to legs and (c) age between 18 to 60 years. Exclusion criteria were, (a) CLBP due to organic pathology in the spine such as malignancy (primary or secondary), or chronic infections checked by X ray of lumbar spine.[16] The study was approved by the institutional review board and the ethical committee of the university. A signed informed consent was obtained from all patients.

### Study design

In this randomized control study, 80 subjects who satisfied the inclusion criteria were allotted to two groups by a computer generated random number table (www.randomizer.org). Numbered opaque envelopes were used to implement the random allocation to conceal the sequence until interventions were assigned. Magnetic resonance Imaging (MRI) scans of all patients were reviewed and X-ray pictures of lumbar spine (antero posterior and lateral view) were obtained. Demographic details vital clinical data, personal, family and stress history were documented before starting the intervention. Outcome variables were recorded on the first and seventh day. The experimental group trained under the yoga-based program whereas the control group received physical therapy exercise based program. The control group went on to the yoga group in the second week. Both groups had the same daily routine with matched intervention [Table 1].

**Table 1:** Time table for two groups for week-long residential program daily schedule of practices for yoga and control group

Time	Yoga group	Control group
05.00-05.30 am	OM meditation – 30 minutes	Walking - 30 minutes
05.30-06.30 am	Yoga based special technique – 60 minutes	Exercise based special technique - 60 minutes
06.30-07.30 am	Bath and wash	Bath and wash
07.30-08.15 am	Chanting of yogic hymns – 45 minutes	Video show (on nature) – 45 minutes
08.15-08.45 am	Breakfast	Breakfast
08.45-10.00 am	Rest	Rest
10.00-11.00 am	Lecture (on yogic lifestyle) – 60 minutes	Lecture (on healthy lifestyle) – 60 minutes
11.00-12.00 noon	Pranayama (yogic breathing) – 60 minutes	Non yogic breathing practice – 60 minutes
12.00-01.00 pm	Yoga based special technique – 60 minutes	Exercise based special technique – 60 minutes
01.00-02.00 pm	Lunch(vegetarian diet)	Lunch(vegetarian diet)
02.00-02.30 pm	Deep relaxation technique – 30 minutes	Rest at room – 30 minutes
02.30-04.00 pm	Assessments and counseling	Assessments and counseling
04.00-05.00 pm	Cyclic meditation – 60 minutes	Listening to music – 60 minutes
06.15-06.45 pm	Divine hymns session (Bhajan) – 30 minutes	Video show (on nature) – 30 minutes
06.45-07.45 pm	Meditation with yogic chants (Mind sound resonance technique) – 45 minutes	Walking – 45 minutes
07.45-08.30 pm	Dinner (vegetarian diet)	Dinner (vegetarian diet)
08.30-10.00 pm	Self study	Self study

Hour to hour matching for the type of practices for the two groups was ensured

**Table 2:** Back pain special techniques for yoga group

I. Supine postures Pavanamuktasana (wind releasing pose) series Supta pawanamuktasana (leg lock pose) Jhulana lurkhanasana (rocking and rolling) Ardha navasana (half boat pose) Uttanapadasana (straight leg raise pose) Sethubandhasana breathing (bridge pose lumbar stretch) Supta udarakarshanasana (folded leg lumbar stretch) Shavaudarakarshanasana (crossed leg lumbar stretch)
II. Prone postures Bhujangasana (serpent pose) Shalabhasana breathing (locust pose)
III. Quick relaxation technique in shavasana (corpse pose)
IV. Sitting postures
III. Quick relaxation technique in shavasana (corpse pose)
IV. Sitting postures Vyaghra svasa (tiger breathing) Shashankasana breathing (moon pose)
V. Standing postures Ardha chakrasana (half wheel pose) Prasarita pada hastasana (forward bend with legs apart) Ardha kati chakrasana (lateral arc pose)
VI. Deep relaxation technique, in shavasana with folded legs

### Blinding and masking

The statistician, who randomized and analysed data, and the researcher who carried out the assessments were blind to the subject’s group. The psychologist scored the coded answer sheets of the questionnaires after completion of the study.

### Intervention

#### Yoga intervention

The specific ‘integrated yoga therapy module for low back pain’ was developed by a team of two yoga experts and a physiatrist. The concepts of the modules were taken from traditional yoga scriptures (p*atanjali yoga sutra, and yoga vasishtha*) that highlight a holistic approach to health management at physical, mental, emotional and intellectual levels.[17] The practices consisted of *asanas* for back pain (yoga postures), *pranayama*, relaxation techniques, meditation, and lectures on yogic lifestyle, devotional sessions and stress management through yogic counseling. The physical practices (back pain special techniques [Table 2], included simple yogic movements and maintenance in the final posture of asanas that provide stretch and relaxation. The safety of the practices was ensured by avoiding acute forward or backward movements of the spine and jerky movements while designing the module.[18]

*Pranayama* included yogic breathing practices to achieve a slow rhythmic pattern of breathing. The instructions for this included (a) slow down the breath rate, (b) exhalation to be made longer than inhalation, and (c) develop an internal awareness. A prolonged easy, slow exhalation is the safest way to get mastery over the mind.[19]

Meditation, considered to be a part of yoga, (*antaranga* yoga) is a valuable tool to calm down uncontrollable surge of negative emotions. Since most patients with CLBP have a component of psychological stress that may contribute to poor quality of life, meditation and devotional sessions are relevant to correct the problem in a holistic way. Lectures and individual yogic counseling for stress management to bring about a notional correction were focused on ‘happiness analysis’.[20]

This analysis helps the patient (a) cognize the source and pattern of their emotional responses, (b) restore freedom to change the emotional responses to chronic pain and demanding life situations and (c) learn to stay on in the blissful bed of inner silence achieved during all joyful moments of life. Thus the IAYT for back pain had a multidimensional approach to promote positive mood and well being through techniques for physical relaxation, breath manipulation to calm down the mind and counseling sessions for cognitive change.

#### Control intervention

The practices consisted of a set of physical movements (certified by the senior physiatrist – Table 3), non-yogic safe breathing exercises, lectures on scientific information including (a) causes of back pain, (b) stress, QOL and CLBP and (c) benefits of physical exercises. For the experimental group, video shows on animals, plants, nature etc. were used as placebo to engage them during the time when the yoga group had video shows on yoga or going through yogic counseling.

**Table 3:** Control group practices

Standing hamstring stretch Cat and Camel Pelvic tilt Partial curl Piriformis stretch Extension exercise Quadriceps leg raising Trunk rotation Double knee to chest Bridging Hook lying march Single knee to chest stretch Lumbar rotation Press up Curl ups

### Outcome variables

#### Quality of life

WHOQOL-BREF is a standardized comprehensive instrument for assessment of QOL comprising of 26 items and was developed by the WHO. The scale provides a measure of an individual’s perception of quality of life for the four domains: (1) physical health (seven items) (2) psychological health (six items) (3) social relationships (three items) and (4) environmental health (eight items). In addition, it also includes two questions for ‘overall quality of life’ and ‘general health’ facets. The domain scores are scaled in a positive direction (i.e., higher scores denote higher quality of life). The range of scores is 4-20 for each domain. The internal consistency of WHOQOL-BREF ranged from 0.66-0.87 (Chronbach’s alpha co-efficient). The scale has been found to have good discriminant validity.[1] It has good test retest reliability and is recommended for use in health surveys and to assess the efficacy of any intervention at suitable intervals according to the need of the study.

### Perceived stress scale

The perceived stress scale (PSS) measures a global perception of stress response on a continuum from mild to severe.[21] It has 10 items asking the subject to rate how often they have perceived an event in their life over the last month. The measure has good reliability, validity and internal consistency with a Cronbach’s alpha of 88. It has been widely used, is general in nature, and brief. We have used it only for baseline correlations with QOL and not after the intervention as it cannot be administered before one month for retest.

### Straight leg raising test[22]

A goniometer (Anand Agencies, Pune) that has two scales fixed at one end to a compass (calibrated in degrees), was used to measure straight leg raising (SLR). It was placed with the stationary arm parallel to the edge of the table, the moving arm along the lateral midline of the thigh and the axis over the superior half of the greater trochanter. Then the leg was raised passively until the patient reported pain. The angle between the two scales is read on the compass. The procedure is repeated on both sides

Other measurements taken in this group of patients to assess disability, spinal flexibility, severity of pain,[15] anxiety and depression have been reported (under revision) as different publications

### Data extraction

PSS was assessed using a five-point scale (0=never; 4=very often). The scores on four items that were worded in the opposite direction were reverse-scored as per the instructions in the manual. The sum of the scores on all 10 items was the total PSS score.[21]

WHOQOLBref: After scoring, the mean values for total and individual domains were computed. These were multiplied by four to obtain the final score comparable to WHOQOL 100 as indicated in the manual.[1]

### Data analysis

Data was analyzed using SPSS version 10.0. Statistical tests used included Kolmogorov–Smirnov’s test for normality of baseline data, independent samples ‘t’ for baseline matching of the two groups, repeated measures ANOVA for group*time interaction followed by post hoc analysis for within and between group differences and Pearson’s correlation test for correlations.

## RESULTS

**Figure 1:**
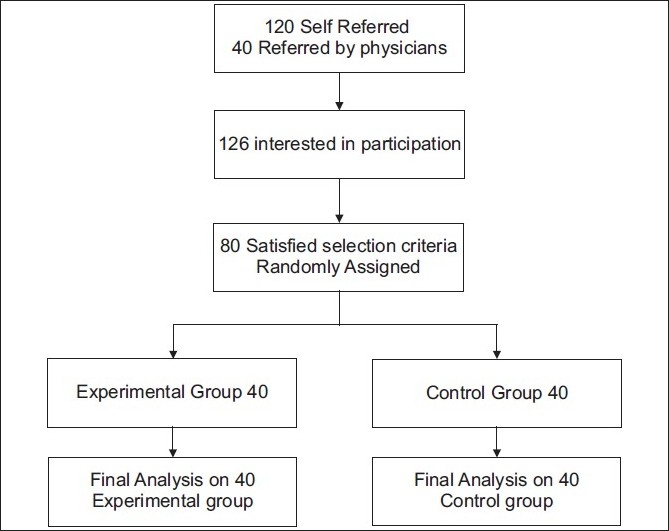
Trial profile

Figure 1 shows the study profile. There were no drop outs as this was a residential short term program. The two groups, yoga and control (40 each), were similar with respect to socio-demographic and medical characteristics [Table 4]. The baseline data for all variables were normally distributed and did not differ significantly between groups (*P*>0.05). Table 5 shows the results of all variables. There were no adverse events.

**Table 4:** Demographic data

Variables	Yoga	Control
Number of participants	40	40
Males (M)	19	25
Females (F)	21	15
Age (mean ± SD)	49 ± 3.6	48 ± 4
Education		
a) High school	M-3, F-11	M-5, F-3.
b) College	M-10, F-8	M-13, F-10
c) Post graduate	M-6, F-2	M-7, F-2
Males		
Working-sedentary	14	16
Working-non sedentary	5	8
Females		
Working	6	7
Housewives	15	8
CLBP		
< 1 year	10	11
1-5 years	9	11
5-10 years	11	10
> 10 years	10	8
Cause		
Lumbar spondylosis (LS)	6	5
Prolapsed intervertebral	6	7
LS with PID	19	15
Muscle spasm	9	13

**Table 5:** Results of all variables post intervention (Repeated measures ANOVA)

		Within groups										Between groups	
		Yoga					Control				
Variable		Mean ± SD	95% CI	ES	% change	*P* values	Mean ± SD	95% CI	ES	% change	*P* values	ES	*P* value
PHY	Pre	11.87 ± 2.5	11.07 to 12.67	1.19	27.55	<0.001	12.49 ± 2.26	11.76 to 13.21	0.25	4.96	0.113	0.253	0.001
	Post	15.14 ± 1.56	14.64 to 15.64				13.11 ± 2.17	12.42 to 13.81					
PSYCH	Pre	13.15 ± 2.34	12.40 to 13.9	0.85	15.82	<0.001	13.12 ± 2.42	12.34 to 13.89	0.08	1.75	0.588	0.085	0.001
	Post	15.23 ± 1.34	14.81 to 15.66				13.35 ± 2.71	12.48 to 14.22					
SOC	Pre	13.43 ± 3.32	12.38 to 14.49	0.43	10.17	0.001	13.50 ± 3.30	12.44 to 14.56	0.47	3.45	0.291	0.60	0.004
	Post	14.80 ± 2.71	13.93 to 15.67				13.03 ± 3.16	12.02 to 14.04					
ENV	Pre	13.45 ± 2.2	12.75 to 14.15	0.54	8.77	0.001	13.44 ± 2.32	12.70 to 14.18	0.03	0.45	0.811	0.036	0.017
	Post	14.63 ± 1.6	14.11 to 15.14				13.50 ± 2.16	12.81 to 14.19					
SLRR	Pre	57.95 ± 20.23	51.48 to 64.42	1.14	31.14	0.001	57.68 ± 24.65	49.79 to 65.56	0.99	18.67	0.001	0.407	0.04
	Post	76.00 ± 16.38	70.76 to 81.24				68.45 ± 20.48	61.90 to 75.00					
SLR L	Pre	59.00 ±18.54	53.07 to 64.93	1.19	28.38	0.001	56.30 ± 23.71	48.72 to 63.88	0.70	21.45	0.001	0.430	0.16
	Post	75.75 ± 15.04	70.94 to 80.56				68.38 ± 19.03	62.29 to 74.46					

PHY = Physical health, PSYCH = Psychological health, SOC = Social health, ENV = Environment, area domains of WHOQOL Bref. SLRR = Straight leg raising right side, SLRL = Straight leg raising left side, CI = Confidence interval, ES = Effect size

### Perceived stress scale

PSS was used only for baseline assessments and correlations with QOL. The mean scores of PSS for the whole group (n=80) was higher (18.80 plus/minus 6.22) than the normative mean value (14.1) for normal adult Indians.[21] There were 60 patients (31 in yoga and 29 in control) who had scores greater than 14. Correlations between scores of PSS and total WHOQOL Bref in the whole group (n=80) showed significant negative correlations at r=0.59, (*P*=0.01). The correlations between PSS and the four domains were as follows. physical r=0.31, (*P*=0.005); psychological r=0.48 (*P*<0.001); social r=0.46 (*P*<0.001) and environmental r=0.39, (*P*<0.001).

## WHOQOL BREF

Table 5 shows the results after the intervention in the two groups. There was significant group*time interaction and difference between groups on all domains at *P*<0.01. Within group analysis showed significant improvement in yoga group and non-significant change in control group on all domains.

### Straight leg raising

There was significant increase in SLR in both groups at *P*<0.001 with significant group*time interaction for SLR right side at *P*=0.04.

## DISCUSSION

This randomized control study on 80 patients with CLBP, who underwent a residential intensive training program for one week, showed that there was significant negative correlation in the baseline values of PSS with all domains of WHOQOL. There was significantly better (*P*<0.01) improvement in quality of life on all domains of WHOQOL and SLR (right) in yoga than control group.

About 75% (60/80) of this group of patients with CLBP had stress levels above 14.1 (limiting value for Indians).[21] The negative correlation observed in this study supports earlier observations of ‘higher the stress levels lower the QOL’ (1). Subjective experience of one’s own life in the light of altered external stressors such as financial strain or family responsibilities[23] can lead to fear of uncertainty of the future[24] resulting in a viscous loop contributing to poor quality of life.[25]

A study on the relationship between chronic pain and health related quality of life (HRQOL) showed that 58% of patients had depression and anxiety with severe reduction in physical, psychological and social well-being.[26] A predictable relationship between HRQOL with functional status and psychological factors has also been documented.[27,28]

Several non-pharmacological interventions including ‘back school program’,[29] mindfulness based meditation,[30] cognitive behavior modification[7] and multidisciplinary programs[31] have been shown to be effective in reducing pain, disability and improving QOL in CLBP. There are no studies on effect of yoga on QOL in CLBP.

Deshpande *et al*. have shown the beneficial effect of a nonresidential integrated yoga program that assessed the QOL using WHO QOL100 on 184 normal volunteers in south India.[32] We have used these scores for comparison with our scores on WHOQOL Bref in CLBP patients.

### Physical health

The baseline scores for this domain (mean 11.9) in CLBP (present study) patients were lower than the scores (mean 13.8) in normal volunteers.[32] After yoga it increased to 14.5 in normal volunteers and 15.14 in patients with CLBP pointing to the normalizing effect of yoga on physical QOL. This domain of WHOQOLBref deals with features such as mobility, fatigue, pain, sleep, work capacity etc The observed improvement can be attributed to reduction in pain and disability with improvement in spinal flexibility.[15] Other studies on integrated yoga in healthy children and adults have shown better physical stamina[33] dexterity and eye hand coordination.[34] Better quality and duration of sleep after yoga has been reported in the elderly.[35]

### Psychological health

There was a significant (20%) improvement in yoga group with non- significant change in control group. The baseline values were much lower (mean 13.0) than normal volunteers (14.7) and increased (15.2 in CLBP) similar to normal volunteers (15.5) after yoga. The improvement seen in this domain that deals with questions relating to feelings, self esteem, spirituality, thinking, learning, memory etc. may be attributed to reduction in anxiety and depression. Several studies have shown the effect of yoga in reducing anxiety[36] depression[37] and stress[38] with enhanced mental health as observed by improved perceptual sharpness,[39] memory[40] and better information processing at the thalamic level.[41]

### Social health

The mean scores changed from 13.0 to 14.8 in CLBP patients and 14.8 to 15.2 in normal volunteers. This domain has questions relating to problems in interpersonal relationships, social support etc which could be the main source of stress contributing to spinal pain. They were addressed during lectures and at a personal level in yoga counseling sessions. They were aimed at achieving an introspective cognitive change by recognizing the psychological freedom ‘to react, not to react or change the usual pattern of reaction to situations’ highlighted in yoga texts.[17] This would have resulted in reversal of the biochemical processes and opened up the connective tissue plasticity.[42]

### Environmental health

The mean value in this domain which was lower (12.8) than normal (14.5) improved significantly to reach normalcy (14.6) after yoga and not after physical exercises. This domain has questions that deal with problems relating to financial resources, physical safety, physical environment such as pollution, noise, climate etc. One of the definitions of yoga (*Bhagavad-Gita*) says that yoga results in equanimity and balance (*samatavam*) that can help in better tolerance to environmental changes (*sheeta ushna samah*-tolerence to heat or cold). Studies have shown that yoga changes the physiological responses to stressors by improving autonomic stability with better parasympathetic tone in normal adults[43] and reducing sympathetic arousal with improved performance in congenitally blind children[44] and community home girls.[45]

Yoga texts explain how integrated yoga techniques help in improving the quality of life. Voluntary reduction in violence and aggressiveness[46] during emotional reactions of anxiety[47] or depression[37] is achieved through restful awareness during all the practices in general and meditation in particular.[48] This mastery over emotional surges leads to controlled and need based physiological responses to stressfully demanding situations instead of uncontrolled overtones of HPA axis[49,50] during chronic pain.

## CONCLUSION

This randomized control study has shown that patients with CLBP had high stress levels with a negative correlation with QOL. A week long residential intensive yoga program increased the QOL and spinal flexibility better than physical therapy exercises for CLBP.

### Limitations of the study include

- Since both groups were in the same campus, the possibility of some interaction and exchange of ideas could not be ruled out, although special care was taken to keep the groups engaged independently
- Short term intervention of one week may be considered a major limitation. A follow-up of patients who were asked to continue the practices daily (one hour) with the help of video and audio instructions is planned

### Suggestions for future work

- Generalization of this program for different cultures needs to be assessed
- Objective measures such as EMG may be included
- Three arm studies that combine yoga during physiotherapy may show synergistic effects
